# The preventive effect of resiniferatoxin on the development of cold hypersensitivity induced by spinal nerve ligation: involvement of TRPM8

**DOI:** 10.1186/s12868-016-0273-8

**Published:** 2016-06-21

**Authors:** Won Uk Koh, Seong-Soo Choi, Ji Hyun Kim, Hye Joo Yoon, Ho-Soo Ahn, Sun Kyung Lee, Jeong Gil Leem, Jun Gol Song, Jin Woo Shin

**Affiliations:** Department of Anesthesiology and Pain Medicine, Asan Medical Center, University of Ulsan, College of Medicine, 88 Olympic-ro 43 gil, Songpa-Gu, Seoul, 05505 Korea

**Keywords:** Hypersensitivity, Resiniferatoxin, Spinal nerve ligation, Transient receptor potential

## Abstract

**Background:**

Resiniferatoxin (RTX) is a potent analog of capsaicin and activates transient receptor potential (TRP) vanilloid type (TRPV) 1. In the current study, we investigated the preventive effect of perineural RTX on the development of cold hypersensitivity induced by spinal nerve ligation (SNL) in rats. Furthermore, we examined the association between the expression level of TRPV1, TRP ankyrin type (TRPA) 1 and TRP melastatin type (TRPM) 8 in the dorsal root ganglion (DRG) and cold hypersensitivity after SNL.

**Results:**

RTX pretreatment prevented the development of SNL-induced hypersensitivity to mechanical, thermal, and cold stimuli. Western blot analysis 4 weeks after RTX pretreatment showed that RTX pretreatment decreased the protein expression level of SNL-induced TRPM8, but not TRPV1 or TRPA1, in the DRG of SNL rats. Immunofluorescent analysis revealed that up-regulated TRPM8-stained neurons after SNL co-localized with neurofilament 200-positive neurons located in the DRG.

**Conclusions:**

Pretreatment with perineural RTX significantly inhibits SNL-induced mechanical, thermal, and cold hypersensitivity. The antinociceptive effect of perineural RTX, especially on cold hypersensitivity, may be related to the suppression of TRPM8 expression in DRG.

**Electronic supplementary material:**

The online version of this article (doi:10.1186/s12868-016-0273-8) contains supplementary material, which is available to authorized users.

## Background

The capsaicin receptor was first cloned in 1997 [1], which was later named the transient receptor potential (TRP) vanilloid type 1 (TRPV1). TRPV1 is a non-selective cation channel sensitive to noxious heat, pH, putative endovanilloids, and pungent plant products such as capsaicin. TRPV1 channels are found in some A-delta and unmyelinated C-fibers of peripheral nerves [2, 3], well known as part of the pain conduction pathway. Therefore, the TRPV1 channel is considered a target for pain control, and many TRPV1 agonists and antagonists are ongoing preclinical and clinical trials as analgesics [4, 5].

Resiniferatoxin (RTX) is a TRPV1 agonist isolated from *Euphorbia resinifera* and an ultrapotent capsaicin analog [6]. It is more potent than capsaicin by 34-fold in its effects on thermoregulation and neurogenic inflammation [6]. Unlike capsaicin, binding of RTX to TRPV1 is irreversible leading to a sustained influx of sodium and calcium through the channels, thereby desensitize the TRPV1 expressing neurons at the dorsal root ganglion (DRG) [7]. Furthermore, RTX has advantages over capsaicin in that it induces less initial irritation and produces less systemic toxicity [8]. RTX has been used for perineural injection like a local anesthetic in animal models, and has been shown to produce conduction analgesia without suppressing motor and other sensory function [9]. Therefore, perineural RTX may hold promise for preventing postoperative pain and consequent chronic neuropathic pain. Indeed, numerous studies revealed that pretreatment with perineural RTX, rather than posttreatment, has a preventive effect on the development of neuropathic pain in various animal pain models. For example, perineural RTX prevents mechanical and heat hypersensitivity after carrageenan injection [10], and in sciatic nerve ligation model of rats, perineural RTX attenuated the development of mechanical and heat hypersensitivity [11, 12]. However, the effectiveness of perineural RTX on preventing cold hypersensitivity is unclear.

Since the characterization of TRPV1, many other families of transient receptor potential channel have been discovered. Among them, transient receptor potential melastatin type 8 (TRPM8) and transient receptor potential ankyrin type 1 (TRPA1) are known as ‘cold sensitive’ channels [13]. These thermo-sensitive channels (TRPV1, TRPM8, and TRPA1) are known to be involved in the development of neuropathic pain, and many researchers have reported that expression of these channels in sensory neurons are increased in various neuropathic pain models [14–20].

In our current study, we investigated the preventive effect of perineural RTX on the development of cold hypersensitivity induced by spinal nerve ligation (SNL) in rats. Furthermore, we examined the association between the expression level of TRPV1, TRPM8, and TRPA1 in the DRG and cold hypersensitivity after SNL.

## Results

### Behavioral tests

**Fig. 1:**
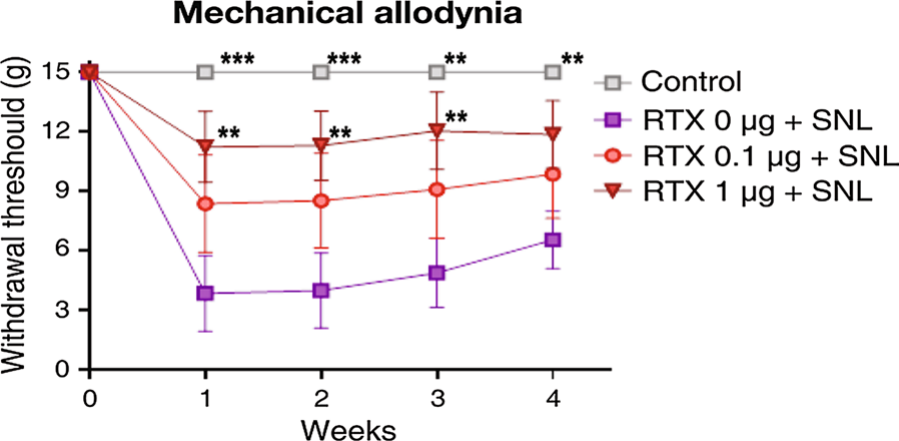
Effect of resiniferatoxin (RTX) pretreatment on the withdrawal threshold to mechanical stimuli. Perineural administration of vehicle or RTX (0, 0.1, or 1 µg) was performed before spinal nerve ligatoin (SNL). Behavioral tests were performed before surgery and weekly for 4 weeks after SNL. To test mechanical hyperalgesia, a von Frey filament was used to stimulate the plantar surface of the ipsilateral left hind foot. The data are presented as the mean ± standard error. **P < 0.01 and ***P < 0.001 compared to the RTX 0 µg group

**Fig. 2:**
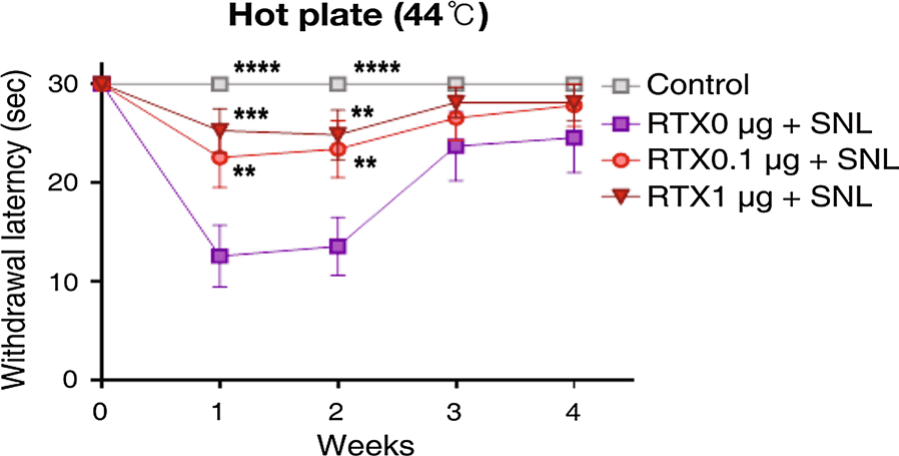
Effect of resiniferatoxin (RTX) pretreatment on the withdrawal latency to heat stimuli. Perineural administration of vehicle or RTX (0, 0.1, or 1 µg) was performed before spinal nerve ligation (SNL). Behavioral tests were performed before surgery and weekly for 4 weeks after SNL. To test heat hyperalgesia, the rats were placed on the 44 °C hot-plate apparatus (Ugo-Basile, Comerico, Italy). The reaction time starting from the placement of the rat on the hot-plate to the time of licking or withdrawing the hind paw was measured. The cut-off time for the hot-plate test was set at 30 s. The data are presented as the mean ± standard error. **P < 0.01, ***P < 0.001 and ****P < 0.0001 compared to the RTX 0 µg group

**Fig. 3:**
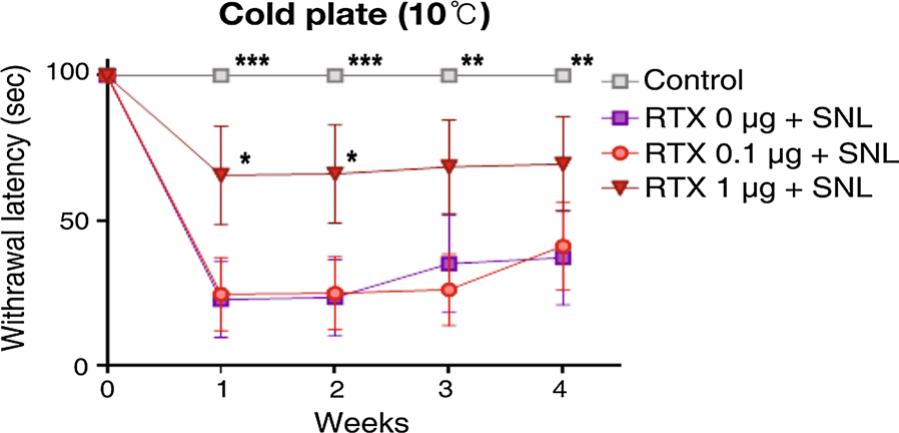
Effect of resiniferatoxin (RTX) pretreatment on the withdrawal latency to cold stimuli. Perineural administration with vehicle or RTX (0, 0.1, and 1 µg) was performed before spinal nerve ligation (SNL). Behavioral tests were performed before surgery and weekly for 4 weeks after SNL. To test cold allodynia, response to cold stimuli was examined using the 10 °C cold-plate (Ugo-Basile, Varese, Italy). The reaction time starting from the placement of the rat on the cold-plate to the time of licking or withdrawing the hind paw was measured. The cut-off time for the cold-plate test was set at 100 s. The data are presented as the mean ± standard error. *P < 0.05, **P < 0.01, ***P < 0.001 and ****P < 0.0001 compared to the RTX 0 µg group

The baseline paw withdrawal threshold to mechanical stimuli in all rats before SNL surgery was 15 g (maximal mechanical stimulus). As shown in Fig. 1, SNL significantly reduced the paw withdrawal threshold to mechanical stimuli after 1 week, and this effect was sustained for 4 weeks after SNL. Perineural administration of RTX 1 µg inhibited the development of SNL-induced mechanical hypersensitivity throughout the whole experimental period, with a significant difference noted compared to the RTX 0 µg group from 1 to 3 weeks after SNL. The paw withdrawal response to heat stimuli (44 °C) is shown in Fig. 2. Compared to baseline values, paw withdrawal latency to heat stimuli significantly decreased from 1 to 2 weeks after SNL. Pretreatment of both RTX 1 µg and 0.1 µg resulted in a significant protective effect against SNL-induced heat hyperalgesia. In the cold-plate test, SNL resulted in significant cold hypersensitivity throughout the whole experimental period (1–4 weeks). In the RTX 1 µg group, the threshold to cold stimuli was higher than in the RTX 0 µg group throughout the experimental period. This protective effect showed statistical significance at 1 and 2 weeks compared to the RTX 0 µg group. In contrast, pretreatment with RTX 0.1 µg did not produce a protective effect on SNL-induced cold hypersensitivity (Fig. 3). The results of the mechanical, cold and hot hypersensitivity are also provided as an additional file (Additional files 1, 2 and 3).

### TRPV1, TRPA1, and TRPM8 protein expression levels in the DRG 4 weeks after SNL

**Fig. 4:**
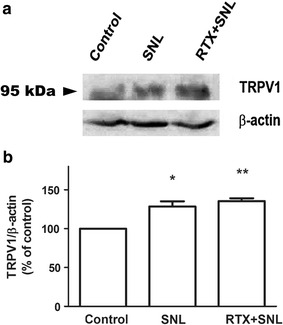
Effect of resiniferatoxin (RTX) pretreatment on transient receptor potential vanilloid type 1 (TRPV1) expression. Perineural administration with vehicle or RTX (0 or 1 µg) was performed before spinal nerve ligation (SNL). To evaluate the protein expression level of TRPV1 located in the dorsal root ganglion (DRG), the left L5 and L6 DRG were isolated 4 weeks after SNL. Western blot analysis was performed using 40 μg of total protein extracted from the isolated DRG (**a**). The specific signal for TRPV1 was quantified and plotted (**b**). β-actin was used as an internal loading control. *P < 0.05, **P < 0.01 compared to the control (sham) group

**Fig. 5:**
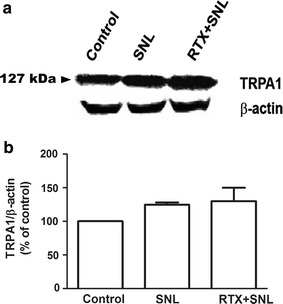
Effect of resiniferatoxin (RTX) pretreatment on transient receptor potential ankyrin type 1 (TRPA1) expression. Perineural administration with vehicle or RTX (0 or 1 µg) was performed prior to spinal nerve ligation (SNL). To evaluate the protein expression level of TRPA1 located in the dorsal root ganglion (DRG), the left L5 and L6 DRG were isolated 4 weeks after SNL. Western blot analysis was performed using 40 μg of total protein extracted from the isolated DRG (**a**). The specific signal for TRPA1 was quantified and plotted (**b**). β-actin was used as internal loading control

**Fig. 6:**
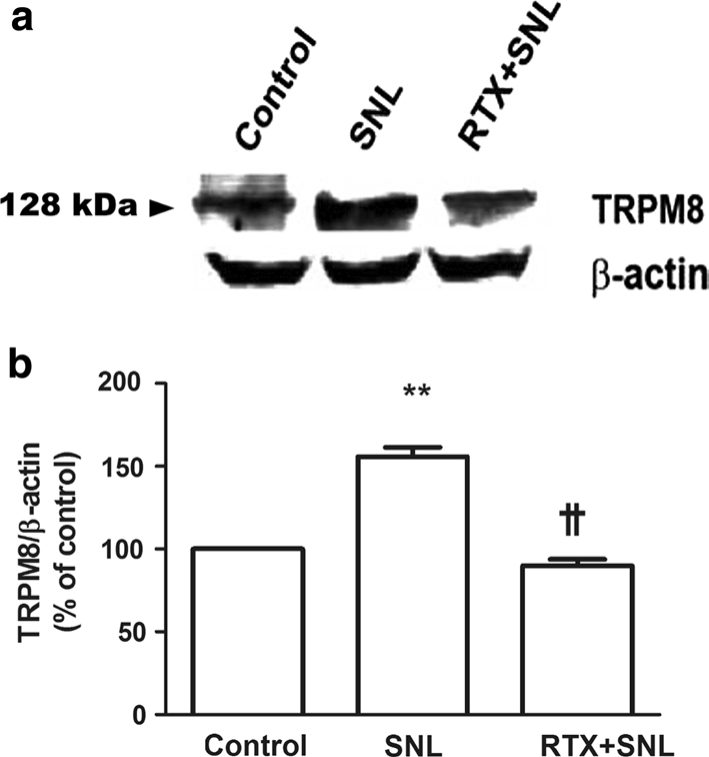
Effect of resiniferatoxin (RTX) pretreatment on transient receptor potential mellastatin type 8 (TRPM8) expression. Perineural administration with vehicle or RTX (0 or 1 µg) was performed before spinal nerve ligation (SNL). To evaluate the protein expression level of TRPM8 located in the dorsal root ganglion (DRG), the left L5 and L6 DRG were isolated 4 weeks after SNL. Western blot analysis was performed using 40 μg of total protein extracted from the isolated DRG (**a**). The specific signal for TRPM8 was quantified and plotted (**b**). β-actin was used as an internal loading control. **P < 0.01 compared to the control (sham) group. ^††^P < 0.01 compared to the SNL group

Immunoblot analysis of the ipsilateral L5 and L6 DRG 4 weeks after SNL revealed that the protein expression level of TRPV1 in the DRG significantly increased 4 weeks after SNL, which was not attenuated by pretreatment with RTX 1 µg (Fig. 4). As shown in Fig. 5, TRPA1 protein expression in the DRG was not affected by either SNL or RTX treatment. On the other hand, TRPM8 protein expression in the DRG significantly increased 4 weeks after SNL and perineural pretreatment with RTX 1 µg before SNL prevented the increase in TRPM8 protein expression (Fig. 6).

### Immunofluorescence analysis of TRPM8 expression

**Fig. 7:**
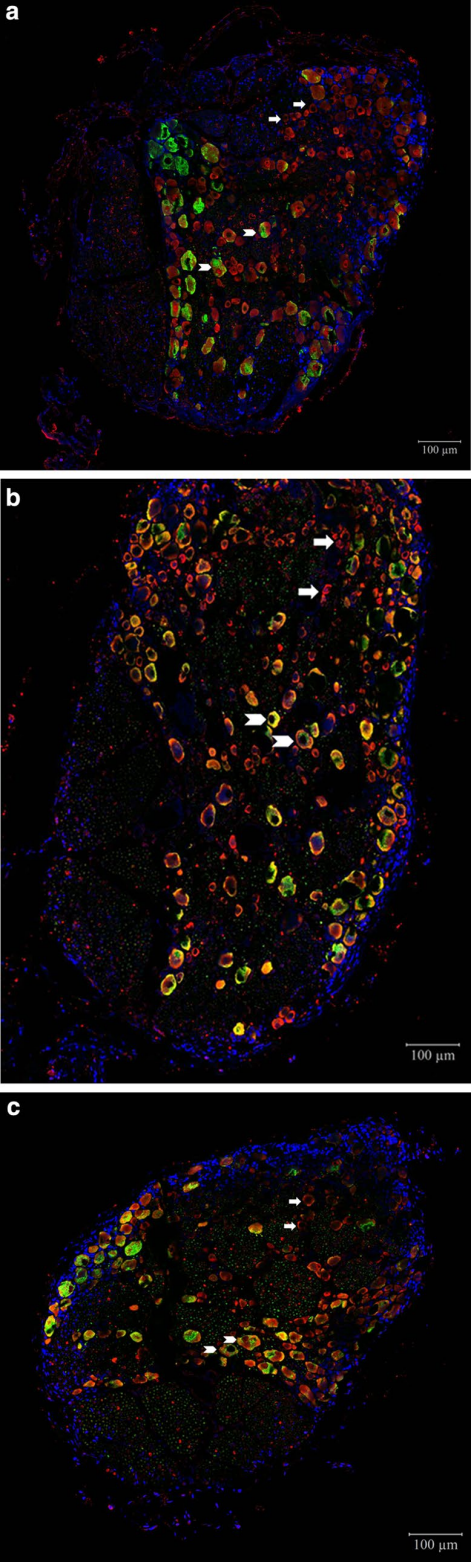
Immunofluorescence view of transient receptor potential mellastatin type 8 (TRPM8) expression after resiniferatoxin (RTX) pretreatment. Perineural administration with vehicle or RTX (0 or 1 µg) was performed before spinal nerve ligation (SNL). To evaluate the protein expression level of TRPM8 located in the DRG, the ipsilateral (*left*) L5 and L6 DRG were isolated 4 weeks after SNL and confocal immunofluorescence analysis was performed. Mounting medium containing 4′,6-diamidino-2-phenylindole (DAPI; *blue*) counterstain was dispersed over the sections before mounting. **a** TRPM8 expression (*red*; *arrows*) in the DRG of sham-operated control group rats. **b** TRPM8 expression (*red*; *arrows*) in the DRG of RTX 0 µg group rats. TRPM8 expression in the DRG was up-regulated and the co-localization of TRPM8 expressing neurons with NF200-positive neurons (*green*) was increased (*arrow heads*). **c** TRPM8 expression (*red*; *arrows*) in the DRG of RTX 1 µg group rats. TRPM8 expression was suppressed compared to that of the RTX 0 µg group

**Fig. 8:**
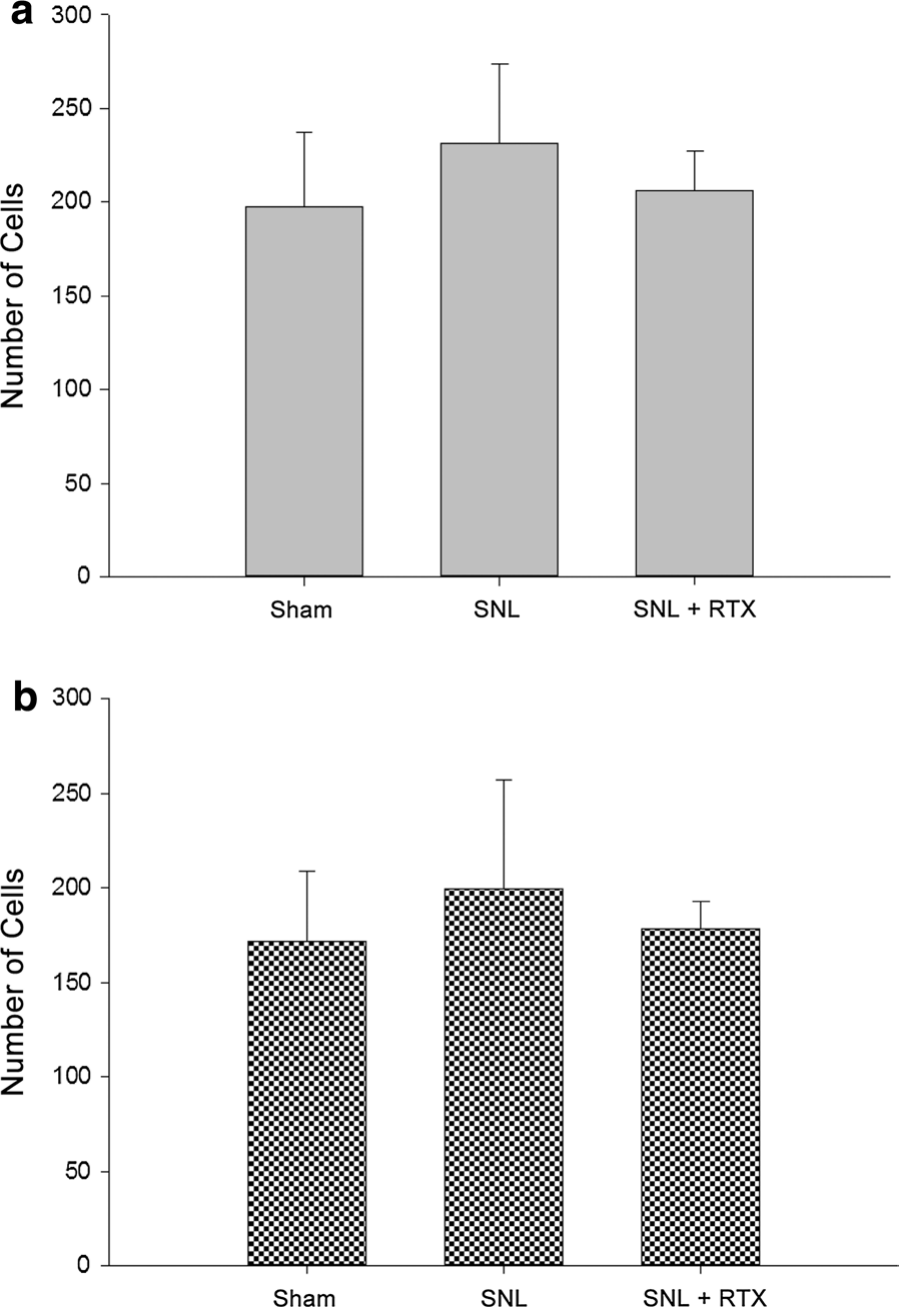
Number of immunoreactive neurons in the ipsilateral L5 dorsal root ganglion (DRG) positive for transient receptor potential melastatin type 8 (TRPM8). Compared with sham operated animals, spinal nerve ligation (SNL) demonstrated an increased trend in number of neurons positive for TRPM8 4 weeks after surgery (**a**). Pretreatment with 1 µg of resiniferatoxin (RTX) decreased the number of neurons presenting TRPM8 compared with the SNL group. The number of neurons coexpressing TRPM8 and neurofilament 200 were also increased in the SNL group compared with the sham group or SNL + RTX group (**b**). Three rats in each group were analyzed

As shown in Figs. 7 and 8, immunofluorescence analysis revealed that SNL surgery (RTX 0 µg group) led to an increased trend in the number of TRPM8-positive neurons in the DRG compared with the sham-operated control group. In the RTX 1 µg group, this increase in the number of TRPM8-expressing neurons was not observed. The results of TRPM8 immunoreactive neuronal cell counts are further provided in additional file 4.

## Discussion

In our current study using a rat model, we found that pretreatment with perineural RTX significantly inhibited mechanical, heat, and cold hypersensitivity after SNL. Western blot analysis demonstrated that TRPV1 and TRPM8 protein expression levels in the DRG had increased 4 weeks after SNL. Perineural pretreatment with RTX reduced the expression level of TRPM8, but not of TRPV1 in the DRG 4 weeks after SNL.

The protective effect of perineural RTX pretreatment on the development of heat and mechanical hypersensitivity has been demonstrated in previous studies using neuropathic models. For example, Kissin et al. reported that pretreatment with perineural RTX reduced the development of mechanical and heat hypersensitivity after carrageenan injection and sciatic nerve ligation [10, 11]. Other authors also reported reduced mechanical and heat hypersensitivity in RTX-pretreated rats after carrageenan injection and common peroneal/tibial nerve ligation [12, 21]. Our findings are consistent with these previous experiments and strengthen the evidence for the protective role of RTX on the development of mechanical and heat hypersensitivity.

Following nerve injury, cold hypersensitivity may develop in addition to mechanical and heat hypersensitivity. Indeed, cold allodynia and hyperalgesia are common symptoms in patients presenting with neuropathic pain. Therefore, the ideal medication to prevent neuropathic pain must have protective effects against cold hypersensitivity along with the protective effects against mechanical and heat hypersensitivity. However, whether perineural RTX exerts a protective effect on cold hypersensitivity remains unclear. Recently, Cruz et al. reported that intraganglionic RTX prevents cold hypersensitivity induced by carrageenan injection into the upper lip and constriction of the infraorbital nerve [22]. In our present study, perineural RTX treatment before nerve injury significantly reduced the development of cold hypersensitivity throughout the entire study period. Taken together, these results indicate that perineural RTX treatment may be an effective method of preventing cold hypersensitivity.

Our experiments focused on the effect and relationship of perineural RTX on the expression of TRP channels after SNL. Various neuropathic pain models have shown that TRPV1 expression in the peripheral nerve increases following nerve injury [15–17, 19]. Nerve injury induces neuropathic pain and especially heat hypersensitivity, which is known to be closely related with increased levels of TRPV1 expression. For instance, in two experiments using an SNL model of rats, ligation of the L5 nerve was associated with increased TRPV1 expression in the L4 DRG [15, 16]. TRPV1 is known to be related not only to the development of heat hypersensitivity but also to mechanical hypersensitivity [21], but the role of TRPV1 in the development of mechanical hypersensitivity is controversial. Previous studies have demonstrated that administration of TRPV1 antagonists in a neuropathic pain model did not attenuate the development of mechanical hypersensitivity [23]. Furthermore, in a paw incision pain model of TRPV1 knockout mice, the development of heat hypersensitivity was suppressed, but in contrast, mechanical hypersensitivity was preserved [24]. However, there are evidences that TRPV1 contributes in the development of mechanical hypersensitivity, especially in the chronic phase. Previous studies demonstrated that antisense oligonucleotide against TRPV1 reduced mechanical hypersensitivity in SNL rats and selective antagonists of TRPV1 attenuated the development of mechanical hypersensitivity [25–27]. Our current study results also showed that TRPV1 expression levels were increased after SNL, which is in agreement with previous study results.

In the present study, we further attempted to elucidate the relationship between cold hypersensitivity and TRP receptors. The TRPM8 is well known to be a cold receptor and a potential transducer for cold temperature mediated allodynia and hyperalgesia. Several studies have shown that TRPM8 expression may be associated with the development of neuropathic pain. For example, Frederick et al. reported that the RNA level of TRPM8 significantly increased after sciatic nerve chronic constriction injury (CCI) [14]. Other reports showed that CCI increased TRPM8 expression in the DRG [18, 20]. Furthermore, nocifencive behavior of mice to cold stimuli has been shown to be related to TRPM8 expression [28, 29]. In our study results, the TRPM8 expression was increased after SNL observed through both Western blot analysis and immunofluorescence analysis. This increase of TRPM8 expression was prevented by RTX pretreatment. Therefore, together with our present results, the preventive effects of perineural RTX on SNL-induced cold hypersensitivity appear to be closely related to the expression of TRPM8 in the DRG. Another interesting finding was that increased co-localization of TRPM8 and NF 200 positive myelinated neurons were detected after SNL surgery. Previous study by Ji et al. reported that in the spinal nerve ligated neuropathic rat presenting cold hypersensitivity, the number of small myelinated A-delta fibers were significantly increased and the number of C-fibers were relatively constant in the plantar skin of the affected hind limb [30]. The authors have concluded that the A-delta fibers provide major contribution in the development of cold hypersensitivity in neuropathic pain. Although the results in this study were electrophysiological quantification of the A-delta and C fibers in the peripheral nerve, the results of this study support our findings of increased expressions of TRPM8 in the myelinated neurons at the DRG in the SNL group demonstrating cold hypersensitivity. The result of our study gives further information that the A-delta fibers presenting TRPM8 may regulate cold hypersensitivity in neuropathic rats.

In contrast to TRPM8, the expression level of TRPA1, another cold receptor, in the DRG did not differ between the three groups at 4 weeks after SNL. In fact, the association between the expression level of TRPA1 and cold hypersensitivity remains controversial. First, many previous studies suggested that TRPA1 is related to the development of cold allodynia and hyperalgesia [14, 31–33], which is not supported by our current data. This discrepancy may be explained by the time course of TRPA1 expression. In a previous study of a sciatic nerve injury model in rats, TRPA1 RNA levels in the DRG increased 1 and 2 weeks after nerve injury, while TRPM8 RNA levels increased 2 weeks after nerve injury [14]. In our present study, the TRPA1 protein level in the DRG was only measured 4 weeks after SNL, while the expression level of TRPA1 at 1–3 weeks were not investigated. Second, some studies have reported inconsistent results on the association between TRPA1 and cold hypersensitivity. For example, one study using TRPM8- and TRPA1-knockout animals reported that noxious cold signaling may not be related to TRPA1 [29]. The authors showed that nocifencive behavior to cold stimuli was absent in TRPM8-knockout and TRPM8/TRPA1-double-knockout mice, but were retained in TRPA1-null mice. Our present study provides evidence that TRPM8 expression, but not TRPA1 expression, is related to cold hypersensitivity at 4 weeks after SNL.

## Conclusions

In conclusion, pretreatment with perineural RTX inhibits the development of SNL-induced mechanical, heat, and cold hypersensitivity. The antinociceptive effect of perineural RTX against cold stimuli seems to be related to the TRPM8 expression in the DRG.

## Methods

This study was reviewed and approved by the Institutional Animal Care and Use Committee (IACUC) of the Asan Institute for Life Sciences, Asan Medical Center. The committee follows the guidelines of the Institute of Laboratory Animal Resources (ILAR). Behavioral testing and analgesiometry were performed according to the ethical guidelines set by the International Association for the Study of Pain (IASP) [34], and the animals were euthanized after completion of planned tests.

### Experimental animals

Male Sprague–Dawley rats (160–180 g) were housed three per cage in a room maintained at 21 ± 1.0 °C with an alternating 12 h light–dark cycle. Water and food were provided ad libitum and permitted to acclimate for at least 3 days prior to experiments. The animals were allowed to adapt to the laboratory environment for at least 2 h before testing, and they were used only once. To reduce variation related to diurnal rhythm, all experiments were performed during the light phase of the cycle (10:00–16:00).

### Group assignment and surgical procedure for animal model

The rats were randomly assigned into four groups: (1) control group (no treatment, no SNL, n = 5), (2) RTX 0 µg group (vehicle before SNL, n = 7), (3) RTX 0.1 µg group (0.0001 % RTX 0.1 mL before SNL, n = 7), and (4) RTX 1 µg group (0.001 % RTX 0.1 mL before SNL). Spinal nerve exposure and ligation were performed as previously described [35]. In brief, the rats were anesthetized by intraperitoneal injection of zoletil (12.5 mg) and xylazine (3 mg). An approximately 1.5-cm midline incision above the left lateral lumbar spine was made, and the paravertebral muscles were retracted, thereby partially exposing the L5 and L6 vertebral bodies. The left L5 and L6 spinal nerves were isolated after partial removal of the left L6 transverse process. Vehicle or RTX (Sigma-Aldrich Co., St. Louis, MO) of the above-indicated doses were administered perineurally distal to the DRG and proximal to the formation of the sciatic nerve. The dose of RTX was chosen based on a previous publication [36]. After 30 min, tight ligation of L5 and L6 spinal nerves was done with 6-0 black silk distal to the DRG and proximal to the formation of the sciatic nerve at the site of RTX or vehicle injection.

### Behavioral tests

Behavioral responses to mechanical, heat, and cold stimuli were performed before surgery and weekly for 4 weeks after SNL surgery. Animals were positioned individually in plastic cages with wire-mesh bottoms and were acclimated for at least 20 min. To verify that the animal responses were normal, baseline behavioral tests were performed before SNL surgery.

Mechanical hypersensitivity was tested using a von Frey filament (Stoelting, Wood Dale, IL). The plantar surface of the affected left hind foot was stimulated. Eight calibrated von Frey filaments (0.41, 0.70, 1.20, 2.00, 3.63, 5.50, 8.50, and 15.10 g) were sequentially applied to the plantar surface of the foot (in ascending or descending order) and gently pressed against the foot. Rapid withdrawal or flinching was interpreted as a positive response. In the case of a positive response, the next-lightest filament was tested; if a negative response was observed, the next-heaviest filament was applied. The 50 % withdrawal threshold was determined by using the previously described up-down method [37].

Heat and cold hypersensitivity was assessed using the hot and cold-plate test. To test heat hypersensitivity, the rats were individually placed on a hot-plate apparatus (Ugo-Basile, Comerico, Italy) with the temperature adjusted to 44 ± 0.1 °C [38], and the reaction time starting from the placement of the rat on the hot-plate to the time of first nocifencive behavior (licking or withdrawing the hind paw) was measured. The cut-off time for the hot-plate test was set at 30 s. Response to cold stimuli was examined using a cold-plate (Ugo-Basile, Comerico, Italy) with the temperature adjusted to 10 ± 0.1 °C [39]. The reaction time starting from the placement of the rat on the cold-plate to the time of licking or withdrawing the hind paw was measured and the cut-off time for the cold-plate test was set at 100 s. The latency to first nocifensive behavior in each rat was regarded as an index to nociceptive threshold. Each rat was tested three times with sufficient intervals between trials to avoid possible effects of anesthesia or tissue damage.

### Total cellular protein extraction and western blot analysis

To evaluate the protein expression levels of TRPV1, TRPA1, and TRPM8 in the DRG, the left L5 and L6 DRG was isolated 4 weeks after SNL, immediately after the last behavioral examination. After dissecting the left L5 and L6 DRG, DRG samples were washed twice with cold Tris buffered saline (TBS; 20 mM Trizma base and 137 mM NaCl, pH 7.5). Immediately after washing, the cells were lysed with sodium dodecyl sulfate (SDS) lysis buffer (62.5 mM Trizma base, 2 % w/v SDS, 10 % glycerol) containing 0.1 mM Na_3_VO_4_, 3 mg/mL aprotonin, and 20 mM NaF. Sonication was performed in brief to reduce viscosity and shear DNA. Each protein concentration was determined with the detergent compatible protein assay reagent (Bio-Rad Laboratories, Richmond, CA) using bovine serum albumin as the standard. Dithiothreitol (5 mM) and bromophenol blue (0.1 % w/v) were further added and boiled. The total protein samples (40 μg) were separated by electrophoresis in 8–10 %-polyacrylamide gels, and transferred onto a polyvinylidene difluoride membrane (Amersham Pharmacia Bioscience, Little Chalfont, Buckinghamshire, UK). The membranes were immunoblotted with antibodies against TRPV1 (1:1000; ab31895; Abcam, Cambridge, MA), TRPA1 (1:1000; ab68847; Abcam), TRPM8 (1:1000; ab104569; Abcam), or β-actin (1:1000; A300-491A; Bethyl Laboratories INC., Montgomery, TX) and visualized with enhanced chemiluminescence (ECL) -plus solution (Amersham Pharmacia Bioscience). The membranes were then exposed to Hyperfilm-MP (Amersham Pharmacia Bioscience) for the detection of light emission. The specific signals for TRPV1, TRPM8, TRPA1, and β-actin were quantified using the ImageJ freeware (NIH).

### Immunofluorescence analysis of TRPM8 expression

Confocal immunofluorescence was performed to detect immunoreactivity in the DRG of SNL rats. TRPM8 and neurofilament 200KDa (NF200; a marker of myelinated neurons) expression levels were analyzed using three rats in each group (total 9 rats) that were not used for behavioral testing in each of the following groups: control group, the RTX 0 µg group, and the RTX 1 µg group. Four weeks after SNL, the animals were anesthetized by intraperitoneal injection of zoletil (12.5 mg) and xylazine (3 mg), and a fixative containing 4 % buffered paraformaldehyde was perfused through the left ventricle. The left L5 DRG was isolated and fixed immediately in the same solution. The tissue samples were embedded in paraffin, and the blocked sections were cut in 10-µm-thick cross sections by the middle of the DRG with a microtome and then mounted onto slides. The sections were deparaffinized with xylene and rehydrated sequentially with 100, 95, and 70 % ethanol and then rinsed twice in distilled water. The slides were then immersed in 0.01 M sodium citrate buffer (pH 6.0) and cooled in air. The sections were blocked with 5 % normal donkey serum, 0.3 % Triton X-100, and 1 % bovine serum albumin (BSA) in phosphate buffered saline-Tween (PBS-T) for 1 h. The sections were incubated overnight at 4 °C with rabbit polyclonal antibodies to TRPM8 (1:1000; ab104569; Abcam) with the IgG fraction of mouse polyclonal antibody to NF200 (1:1000; n0142; Sigma, St Louis, MO). The sections were subsequently incubated with Alexa Flour 546 donkey anti-rabbit IgG (red; 1:1000; Invitrogen, Carlsbad, CA) and Alexa Flour 488 donkey anti-mouse IgG (green; 1:1000; Invitrogen). The sections were rinsed in PBS-T and 25 μL of mounting medium containing the 4′,6-diamidino-2-phenylindole (DAPI; H-1200; Vector; Burlingame; CA) counterstain was dispersed over the sections and then mounted onto slides. Immunofluorescence was assayed using a confocal fluorescent microscope (Olympus BX51 system microscope, Tokyo, Japan) with imaging software (Image Pro Plus ver.5.1, Media Cybernetics, Rockville, MD). The number of immunoreactive cells was counted manually in three slices of the DRG for each group. The total number of TRPM8 expressing neurons and doubly labeled neurons were counted and compared between each group.

### Statistical analysis

The data are presented as the mean ± standard error (SEM). Behavioral data (withdrawal threshold to mechanical, thermal, and cold stimuli) were evaluated by two-way repeated measures analysis of variance (ANOVA). The statistical significance of differences for the quantified specific signals for TRPV1, TRPM8, and TRPA1 was assessed with one-way ANOVA followed by the Bonferroni’s post hoc test. P values less than 0.05 were considered statistically significant.

## Additional files

10.1186/s12868-016-0273-8 The data of the results of behavioral test: mechanical hypersensitivity.
10.1186/s12868-016-0273-8 The data of the results of behavioral test: cold hypersensitivity.
10.1186/s12868-016-0273-8 The data of the results of behavioral test: hot hypersensitivity.
10.1186/s12868-016-0273-8 The data of TRPM8 immunoreactive neuronal cell count.

## Abbreviations

BSA: bovine serum albumin
DRG: dorsal root ganglion
ECL: enhanced chemiluminescence
IgG: immunoglobulin G
PBS-T: phosphate buffered saline-Tween
RTX: resiniferatoxin
SDS: sodium dodecyl sulfate
SEM: standard error measure
SNL: spinal nerve ligation
TBS: tris buffered saline
TRPV1: transient receptor potential vanilloid type 1
TRPA1: transient receptor potential ankyrin type 1
TRPM8: transient receptor potential melastatin type 8
