# Supplementary material for: The preventive effect of resiniferatoxin on the development of cold hypersensitivity induced by spinal nerve ligation: involvement of TRPM8
Source: BMC Neurosci. 2016 Jun 21;17:38. doi: 10.1186/s12868-016-0273-8 (PMC4915067; doi:10.1186/s12868-016-0273-8)
Supplement: Supplementary file 1 — 10.1186/s12868-016-0273-8 The data of the results of behavioral test: mechanical hypersensitivity. [file 12868_2016_273_MOESM1_ESM.pdf]

| TRPM8 | sham | SNL | RTX |
|---|---|---|---|
| | 237 | 202 | 230 |
| | 158 | 280 | 189 |
| | 198 | 211 | 200 |

| TRPM8 + NF200 merged cells | sham | SNL | RTX |
|---|---|---|---|
| | 209 | 156 | 195 |
| | 135 | 265 | 168 |
| | 171 | 178 | 172 |
