# Supplementary material for: The preventive effect of resiniferatoxin on the development of cold hypersensitivity induced by spinal nerve ligation: involvement of TRPM8
Source: BMC Neurosci. 2016 Jun 21;17:38. doi: 10.1186/s12868-016-0273-8 (PMC4915067; doi:10.1186/s12868-016-0273-8)
Supplement: Supplementary file 2 — 10.1186/s12868-016-0273-8 The data of the results of behavioral test: cold hypersensitivity. [file 12868_2016_273_MOESM2_ESM.pdf]

Cold hypersensitivity

	Time(Week)/Animal(no.)	1	2	3	4
control group					
	0	100	100	100	100
	1	100	100	100	100
	2	100	100	100	100
	3	100	100	100	100
	4	100	100	100	100
RTX 0 ug + SNL	0	100	100	100	100
	1	5	7	3	11
	2	6	7	5	11
	3	9	7	6	14
	4	9	8	9	19
RTX 0.1 ug + SNL	0	100	100	100	100
	1	10	9	12	100
	2	12	9	13	100
	3	17	10	12	100
	4	23	100	15	100
RTX 1 ug + SNL	0	100	100	100	100
	1	100	6	100	45
	2	100	5	100	49
	3	100	7	100	61
	4	100	6	100	69

5	6	7
100		
100		
100		
100		
100		
100	100	100
8	100	25
8	100	27
9	100	100
15	100	100
100	100	100
16	11	14
15	13	12
17	15	12
20	17	13
100	100	100
100	8	100
100	8	100
100	11	100
100	11	100
