# Supplementary material for: The preventive effect of resiniferatoxin on the development of cold hypersensitivity induced by spinal nerve ligation: involvement of TRPM8
Source: BMC Neurosci. 2016 Jun 21;17:38. doi: 10.1186/s12868-016-0273-8 (PMC4915067; doi:10.1186/s12868-016-0273-8)
Supplement: Supplementary file 3 — 10.1186/s12868-016-0273-8 The data of the results of behavioral test: hot hypersensitivity. [file 12868_2016_273_MOESM3_ESM.pdf]

Hot hypersensitivity

control group	Time(Week)/Animal(no.)		1	2	3	4
		0	30	30	30	30
		1	30	30	30	30
		2	30	30	30	30
		3	30	30	30	30
		4	30	30	30	30
RTX 0 ug + SNL		0	30	30	30	30
		1	7	9	9	10
		2	9	10	8	11
		3	9	12	30	30
		4	10	12	30	30
RTX 0.1 ug + SNL		0	30	30	30	30
		1	7	17	24	30
		2	9	18	23	30
		3	14	28	24	30
		4	15	30	30	30
RTX 1 ug + SNL		0	30	30	30	30
		1	30	20	30	17
		2	30	21	30	15
		3	30	28	30	30
		4	30	30	30	30

5	6	7
30		
30		
30		
30		
30		
30	30	30
16	30	7
17	30	10
25	30	30
30	30	30
30	30	30
25	26	29
27	30	27
30	30	30
30	30	30
30	30	30
30	20	30
30	18	30
30	19	30
30	17	30
