# Supplementary material for: The preventive effect of resiniferatoxin on the development of cold hypersensitivity induced by spinal nerve ligation: involvement of TRPM8
Source: BMC Neurosci. 2016 Jun 21;17:38. doi: 10.1186/s12868-016-0273-8 (PMC4915067; doi:10.1186/s12868-016-0273-8)
Supplement: Supplementary file 4 — 10.1186/s12868-016-0273-8 The data of TRPM8 immunoreactive neuronal cell count. [file 12868_2016_273_MOESM4_ESM.pdf]

Mechanical hypersensitivity

	Time(Week)/Animal(no.)		1	2	3	4
control group		0	15	15	15	15
		1	15	15	15	15
		2	15	15	15	15
		3	15	15	15	15
		4	15	15	15	15
RTX 0 ug + SNL		0	15	15	15	15
		1	0.876	0.876	1.556	1.556
		2	1.648	0.876	1.556	1.556
		3	4.339	4.339	1.556	1.556
		4	4.339	5.607	4.339	5.362
RTX 0.1 ug + SNL		0	15	15	15	15
		1	1.556	1.556	1.556	11.98
		2	1.648	1.556	2.56	11.98
		3	1.846	2.248	2.649	15
		4	2.805	4.339	3.708	15
RTX 1 ug + SNL		0	15	15	15	15
		1	11.98	6.908	2.805	11.98
		2	11.98	7.432	2.805	11.98
		3	15	6.738	2.592	15
		4	15	6.738	4.339	15

5	6	7
15		
15		
15		
15		
15		
15	15	15
2.805	15	4.331
2.978	15	4.331
3.117	15	4.331
4.467	15	6.738
15	15	15
11.98	15	15
11.98	15	15
11.98	15	15
13.22	15	15
15	15	15
15	15	15
15	15	15
15	15	15
11.98	15	15
